# ATP6V0A1 protects dopaminergic neurons via the autophagy–lysosomal pathway in Parkinson’s disease

**DOI:** 10.4103/NRR.NRR-D-24-01420

**Published:** 2025-06-19

**Authors:** Yuwan Lin, Zixin Tan, Wenfeng Ye, Weimin Li, Hao Chen, Yuping Lin, Miaomiao Zhou, Hanqun Liu, Qin Liu, Zhiling Zhang, Weixin Kong, Zongtang Xu, Hao Lin, Mingshu Mo, Wenyuan Guo, Keye Lin, Jiaxin Tang, Yueying Zheng, Wenlong Zhang, Pingyi Xu, Xiang Chen

**Affiliations:** 1 Department of Neurology, the First Affiliated Hospital of Guangzhou Medical University, Guangzhou, Guangdong Province, China; 2 Department of Neurology, Shanghai General Hospital, Shanghai Jiao Tong University School of Medicine, Shanghai, China; 3 Department of Geriatrics, Guangzhou First People’s Hospital, Guangzhou, Guangdong Province, China; 4 Department of Neurology, Guangzhou Medical University, Guangzhou, Guangdong Province, China

**Keywords:** ATP6V0A1, autophagy, dopaminergic neurons, lysosome, mTORC, Parkinson’s disease, vacuolar H^+^-ATPase, α-synuclein

## Abstract

Parkinson’s disease is the second most common neurodegenerative disorder. ATPase H^+^ transporting V0 subunit A1 (ATP6V0A1) is a component of vacuolar H^+^-ATPase (V-ATPase), an ATP-dependent proton pump. Our previous research identified an association between the ATP6V0A1 rs601999 variant and Parkinson’s disease; however, the underlying mechanisms of ATP6V0A1 in Parkinson’s disease remain elusive. In this study, we generated ATP6V0A1 knockdown and overexpression models and then examined the degeneration of dopaminergic neurons, lysosomal function, and the autophagy–lysosomal pathway using immunohistochemistry, western blotting, and transmission electron microscopy. We found that ATP6V0A1 protected against lysosomal dysfunction, regulated autophagic flux, and decreased phosphorylated α-synuclein levels *in vitro*. *In vivo*, ATP6V0A1 reduced levels of α-synuclein and phosphorylated α-synuclein proteins, mitigated degeneration of dopaminergic neurons, and improved motor dysfunction. Collectively, these findings show that ATP6V0A1 plays a protective role in Parkinson’s disease by modulating the autophagy–lysosomal pathway. A correlation between ATP6V0A1 and Parkinson’s disease susceptibility may serve as a biomarker for Parkinson’s disease, while the protective effects of ATP6V0A1 could represent a potential therapeutic target for the disease.

## Graphical abstract

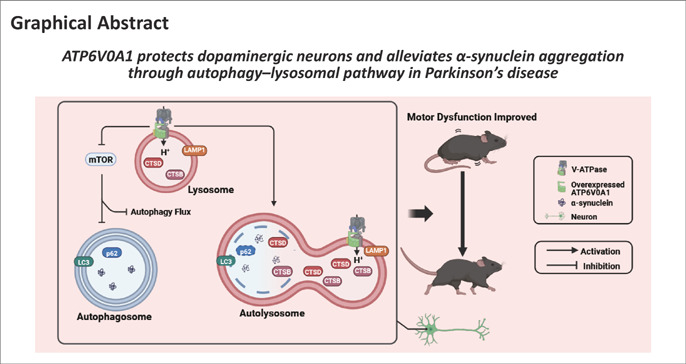

## Introduction

Parkinson’s disease (PD) is characterized by dopaminergic (DA) neurodegeneration and α-synuclein (α-syn) aggregation in the substantia nigra (SN) (Guo et al., 2020; Horsager et al., 2020; Ishikawa, 2023; Ni, 2025). As a neurodegenerative disease, PD is becoming more prevalent in older adults, affecting approximately 1% of individuals over 60 years of age (Tanner et al., 2014; Xu et al., 2016; Tysnes and Storstein, 2017). Despite these insights, the complex interplay between aging, genetic susceptibility, and environmental factors means that the exact etiology of PD remains elusive (Bogers et al., 2023; Cherian et al., 2023; Costa et al., 2023). Around 5% to 10% of PD cases are inherited as familial PD, demonstrating the critical role that genetic vulnerability plays in the development of PD (Seol, 2010). In this regard, synuclein alpha (SNCA), leucine-rich repeat kinase 2 (LRRK2), glucocerebrosidase, and parkin are highly relevant genes (Blauwendraat et al., 2020; Polissidis et al., 2020; Pal et al., 2023). Moreover, our previous research has shown that the ATPase H^+^ transporting V0 subunit A1 (ATP6V0A1) variant, rs601999, is associated with risk of PD (Chen et al., 2019); however, the molecular mechanisms underlying ATP6V0A1 involvement in PD remain unknown.

Vacuolar H^+^-ATPase (V-ATPase) is an ATP-dependent proton pump consisting of a cytoplasmic V1 domain and a transmembrane V0 domain that facilitates proton transport across the membranes of various organelles, especially lysosomes and endosomes. It is therefore crucial for vesicular acidification and closely related to autophagy (Santos-Pereira et al., 2022; Satarker et al., 2022). Autophagy is a vital physiological activity, and its dysfunction contributes to α-syn deposition and DA neurodegeneration (Qin et al., 2021; Nechushtai et al., 2023; Themistokleous et al., 2023). ATP6V0A1, the a1 subunit of the V0 domain, is abundantly expressed in neurons and essential for brain physiological function (Schulz et al., 2007). Reduced ATP6V0A1 expression in a presenilin 1/amyloid beta protein precursor transgenic model of Alzheimer’s disease (AD) results in lysosomal acidification defects, autophagy impairment, and pathogenic protein accumulation (Lee et al., 2010). In microglia, ATP6V0A1 is primarily distributed in early and late phagosomes, with defects including impaired transition from early to late phagosomes (Chen et al., 2024a). Nevertheless, the role of ATP6V0A1 in autophagy in PD remains elusive.

Considering that the role of ATP6V0A1 in PD remains unclear, we investigated the underlying mechanisms of ATP6V0A1 both *in vitro* and *in vivo*.

## Methods

### Cell culture

DA neurons are predominantly expressed in the SN, making them a key focus in the study of PD (Mallet et al., 2019). MN9D cells (HTX1982), which are DA neurons derived from the mouse midbrain, are frequently used as a model for PD research. The MN9D neuronal cell line was purchased from Otwo Biotech Inc. (Cat# HTX1982, RRID: CVCL_M067, Shenzhen, China). Cells were grown in high-glucose Dulbecco’s Modified Eagle Medium supplemented with 10% fetal bovine serum (Gibco, Grand Island, NY, USA). The medium was augmented with 1% penicillin (10,000 U/mL) and streptomycin (10,000 μg/mL) (Gibco). The culture incubator operated at 37°C with a 5% CO_2_ concentration.

### Animals

Men are twice at risk of developing PD compared to women; however, female patients experience a relatively higher mortality rate and a more rapid progression of the disease. Estrogen has also been shown to exert a protective effect on DA neurons. Thus, male C57BL/6 mice were used for these experiments (Jurado-Coronel et al., 2018; Philipe et al., 2022). Male C57BL/6 mice at 8 weeks old (23–25 g) were purchased from Spafas Biotechnology Co., Ltd. (Beijing, China) (license No. SYXK (Jing) 2024-0010). The genetic background of the mice was C57BL/6 with a wild-type genotype. The human (h)-SNCA*A53T mutation mice were obtained from Guangdong Kasee Biotechnology Co., Ltd. (license No. SCXK (Yue) 2024-0069). The genetic background of the mice was C57BL/6 with a homozygous genotype for the hSNCA*A53T mutation. These mice were established by inserting human A53T missense mutants of SNCA cDNA into the SNCA downstream promoter. Both types of mice were sacrificed at 12 months old. During the experimental process, relevant national and institutional animal protection regulations (such as the Regulation on the Management of Experimental Animals) were adhered to, ensuring that the living environment of all animals met the necessary standards. All mice were housed in a specific pathogen-free environment at a density of five mice per cage. All mice were acclimatized for one week in a controlled environment of 22 ± 1°C and approximately 60% ± 5% humidity, following a 12-hour light/dark cycle. Food and water were available *ad libitum*. All experimental protocols were performed in accordance with the National Institutes of Health Guide for the Care and Use of Laboratory Animals (8^th^ ed., National Research Council, 2011). During experiments, mice were administered 0.6% pentobarbital (Smart, Guangzhou, China) to minimize their suffering and stress. The Ethics Committee of the First Affiliated Hospital of Guangzhou Medical University approved the animal experiments (approval Nos. 2022281 and 2022285) on June 9, 2022 and June 15, 2022. Experimental flowchart and timeline are shown in **Additional Figures 1** and **2**.

### Mouse experiments

The α-syn overexpression mouse model is primarily used to investigate α-syn propagation, clearance, and aggregation. The effect of ATP6V0A1 on α-syn aggregation was examined using an α-syn deposition PD model in male C57BL/6 mice. Controls were administered a hSyn promoter-EGFP-MCS-SV40 PolyA (GV503) overexpression vector. While in the experimental group, adeno-associated viral (AAV) ATP6V0A1 at a dose of 1.47 × 10^13^ vg/mL was stereotaxically injected into the SN along with a human α-syn overexpression AAV (WZ Biosciences Inc., Jinan, China), in accordance with Paxinos and Franklin’s stereotaxic coordinates of the mouse brain (Cecyn and Abrahao, 2023). For the control group, AAV-GFP at a dose of 1.08 × 10^13^ vg/mL was administered. The mice were kept in the same setting on a cycle of 12 hours each of light and darkness. Six weeks after injection, the mice were sacrificed. Samples from the striatum and midbrain were gathered for additional examination.

### Cell transfection

For ATP6V0A1 short hairpin (sh)-RNA knockdown, three shRNAs targeting ATP6V0A1 sequence were obtained based on the lentiviral vector pLKD-CMV-Neo-U6-shRNA (Obio Technology Corp., Ltd., Shanghai, China). The shRNA sequences were: shRNA-1, 5′-CCT CCA AAT GGT TCT GAA T-3′; shRNA-2, 5′-CCT GGA CTC CAT CCA GTT T-3′; and shRNA-3, 5′-GCA CGA GAA TGA GAT GTT T-3′. For ATP6V0A1 overexpression and A53T mutation α-syn overexpression, lentiviruses were constructed using the Ubi-MCS-MYC-SV40-Neomycin vector and Ubi-MCS-3FLAG-SV40-puromycin vector, respectively (Genechem Co., Ltd., Shanghai, China).

The impact of ATP6V0A1 on α-syn aggregation was determined by examining α-syn pathological protein levels in an α-syn overexpression model. The PD cell model was generated by transfection with α-syn lentivirus. After α-syn model construction, ATP6V0A1 or ATP6V0A1 shRNA adenovirus was transfected to create a double transfection cell line. Controls were transfected with the corresponding empty vectors.

For transfection, MN9D cells were seeded into 12-well plates at a density of approximately 2 × 10^5^ cells per well. Cells were transduced with either A53T lentivirus or its vector at a multiplicity of infection of 100, depending on the lentiviral titer. After culturing for 24 hours, the cell medium was changed and cells cultured for an additional 48–72 hours. This was followed by treatment with puromycin or neomycin for 3–5 days to screen for stably transfected cells. The transfection efficacy was determined at the protein or RNA level by western blotting or real-time PCR, respectively. Transfected cells were also transduced with ATP6V0A1 shRNA or ATP6V0A1 overexpression lentivirus and its vector.

### ATPase activity detection

ATPase activity of MN9D cells was determined using the ATPase/GTPase Activity Assay Kit (Sigma–Aldrich, St. Louis, MO, USA). First, phosphate standards were used, as per the kit instructions. MN9D cells transfected with vector and shATP6V0A1 were collected and assay buffer was added to solubilize the samples. The soluble material was then harvested by centrifugation at 180,999 × *g* for 10 minutes. Next, 10 μL of supernatant was dispensed into duplicate wells of a 96-well plate, with 10 μL of assay buffer added to a separate well as a negative control. Then, a reaction mix comprising 20 μL of assay buffer and 4 mM (10 μL) ATP was administered to each sample and incubated at ambient temperature for 30 minutes. Subsequently, each well received 200 μL of reagent and was incubated for an additional 30 minutes at ambient temperature.

Finally, the enzymatic reaction was terminated. Absorbance was measured at 620 nM, and enzyme activity was calculated based upon the absorbance value. Under the assay conditions, the amount of enzyme catalyzing the production of 1 mmol of free phosphate per minute was one unit. The final ATPase activity was calculated using the following formula:


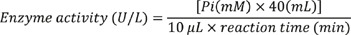


### LysoTracker probes

LysoTracker Red DND-99 (Life Technologies, Gaithersburg, MD, USA) was used to identify lysosome acid fluorescence in MN9D cells. Briefly, MN9D cells were transfected with either of the lentiviral vectors or shATP6V0A1, and then cultured with the appropriate culture medium at a density of 30%–40% in a confocal dish (Wuxi NEST Biotechnology Co., Ltd., Wuxi, China). The original medium was discarded and medium containing 50 nM LysoTracker Red DND-99 (L7528, Invitrogen, Carlsbad, CA, USA) was applied to the cells for 1 hour in a CO_2_ incubator. The staining medium was subsequently replaced, and the cells were rinsed three times with cold phosphate buffered saline (PBS). Cells were then fixed with 1% paraformaldehyde for one hour and then washed a further three times. Finally, the nuclei were stained with 4,6-diamidino-2-phenylindole (DAPI) (F605, Sigma–Aldrich). The samples were observed by microscopy (Leica, Wetzlar, Germany) at an excitation wavelength of 555 nM.

### Lysosome activity

Cathepsin B (CTSB) and cathepsin D (CTSD) activities of control (Ctrl)-shRNA and ATP6V0A1-shRNA cell lines were determined using kits (Abcam, Cambridge, UK). The standards in these kits were measured as indicated in the manufacturer’s instructions. Briefly, MN9D cells transfected with Ctrl-shRNA and ATP6V0A1-shRNA were harvested, washed two times with cold PBS, and resuspended in 1 mL PBS. The cells were then harvested by centrifugation at 4°C at 923 × *g* for 5 minutes, and lysed for 10 minutes in ice-cold cell lysis buffer. Subsequently, lysates were centrifuged at 4°C at 16,000 × *g* for 5 minutes and the supernatants added to a 96-well plate (Corning, Corning, NY, USA). Afterwards, the samples were mixed with 50 μL of reaction buffer and 2 μL of substrate, and reacted at 37°C for 1.5 hours. For measurement of CTSB activity, excitation and emission wavelengths of 400 nM and 505 nM were detected by a fluorescent plate microreader (Biocompare, Austin, TX, USA). CTSD activity was similarly measured at 328 nM and 460 nM.

### RNA sequencing

MN9D cell pellets were lysed in 1 mL TRIzol reagent (Invitrogen) to extract total RNA. Qualified and quantified RNA was measured using a NanoDrop2000/2000c spectrophotometer (Thermo Fisher Scientific, Waltham, MA, USA). Then, mRNA was purified using oligo (dT) adsorbed magnetic beads, and subsequently disassembled into smaller pieces in fragmentation solution. Next, first and second cDNA strands were sequentially synthesized by reverse transcription. The cDNA products were obtained using PCR amplification and further purified by Ampure XP bead adsorption. After dissolving in elution buffer, the cDNA was analyzed on an Agilent 2100 bioanalyzer (Agilent Technologies, Inc., Santa Clara, CA, USA). The final library was obtained after the cDNA was heated and circularized. All sequencing procedures were conducted at BGI Co., Ltd. (Shenzhen, China).

### Bioinformatic analysis

RNA sequencing reads were filtered using SOAPnuke (https://github.com/BGI-flexlab/SOAPnuke) to eliminate reads containing sequence adapters, a high proportion of low-quality bases, or a significant number of unknown sequences. The final data were stored in FASTQ format. The reference genome index was constructed using HISAT2 (http://www.ccb.jhu.edu/software/hisat/index.shtml), and clean reads were corrected according to the comparative gene set of Bowtie2 (v2.2.5) (http://bowtiebio.sourceforge.net/%2520Bowtie2%2520/index.shtml). Then, RSEM (v1.2.12) (https://github.com/deweylab/RSEM) was used to compute related gene levels, and a heatmap was generated via heatmap software (v1.0.8) (https://cran.r-project.org/web/packages/pheatmap/index.html). Gene expression variability between the two groups was determined using DESeq2 (v1.4.5) (http://bioinfo.au.tsinghua.edu.cn/software/degseq/) with *q*-values ≤ 0.05. Additionally, Gene Ontology (GO) and Kyoto Encyclopedia of Genes and Genome enrichment analyses of differentially expressed genes (DEGs) were performed using the Phyper library and hypergeometric test. *P*-values were modified by the Benjamini correction method. DEGs were determined according to the following standard: *q*-value ≤ 0.05 and |log2(fold change)| > 1.

### Protein extraction and western blotting

For total protein extraction, samples involving MN9D cells or animal midbrain tissues were kept on ice and homogenized in radioimmunoprecipitation assay lysis buffer (Beyotime, Jiangsu, China) supplemented with 1% protease inhibitors (Beyotime) and 1% phosphatase inhibitors (MedChemExpress, Shanghai, China) for 15–20 minutes. Then the samples were ultrasonicated. Lysates were centrifuged at 4°C at 12,000 × *g* for 15 minutes. The supernatant concentration was determined by bicinchoninic acid assay (Thermo Fisher Scientific). Samples were then loaded in wells and separated by sodium dodecyl sulfate–polyacrylamide gel electrophoresis (Bio-Rad, Hercules, CA, USA). For cell samples, 40 µg of protein was loaded per well, while 20 µg was loaded for animal samples. After electrophoresis through acrylamide gels, proteins were electroblotted onto polyvinylidene fluoride membranes (Thermo Fisher Scientific) and subsequently blocked with 5% fat-free milk at room temperature for 1–2 hours. Relevant primary antibodies were incubated for 14–16 hours at 4°C. The following day, membranes were washed with Tris-buffered saline containing 0.1% Tween-20 (TBST), three times for 15 minutes each. Subsequently, secondary antibodies were incubated on membranes for 1–2 hours at ambient temperature. Following three more washes with TBST, immunoreactive membranes were detected using chemiluminescence (Yeasen, Shanghai, China). Images were captured using a chemiluminescence gel imaging system (PXi9, Syngene, Cambridge, UK). Target band intensity was calculated using ImageJ software (version 1.8.0, NIH, Bethesda, MD, USA), with -actin (ACTB) serving as the internal control. The antibodies used for western blotting are listed in **Table 1**.

**Table 1:** Antibodies used in this study

Antibody	Dilution	Cat#	RRID	Supplier	Application
Rabbit anti-β-actin	1:20000	AC026	AB_2768234	ABclonal (Wuhan, China)	WB
Rabbit anti-MYC-Tag	1:1000	AE010	AB_2770408	ABclonal	WB
Rabbit anti-ATP6V0A1	1:1000	ab105937	AB_10862106	Abcam	WB
Rabbit anti-LAMP-1	1:1000	ab24170	AB_775978	Abcam	WB
Rabbit anti-α-syn	1:1000	ab138501	AB_2537217	Abcam	WB
Rabbit anti-SOSTM1/p62	1:1000	39749	AB_2799160	Cell Signaling Technology (Danvers, MA, USA)	WB
Rabbit anti-CTSD	1:1000	2284	AB_10694258	Cell Signaling Technology	WB
Rabbit anti-CTSB	1:1000	31718	AB_2687580	Cell Signaling Technology	WB
Rabbit anti-P70 S6 kinase (S6K)	1:1000	2708	AB_390722	Cell Signaling Technology	WB
Rabbit anti-phospho-p70 S6K (Thr389)	1:1000	9234	AB_2269803	Cell Signaling Technology	WB
Rabbit anti-mTOR1	1:1000	2983	AB_2105622	Cell Signaling Technology	WB
Rabbit anti-phospho-mTOR (Ser2448)	1:1000	5536		Cell Signaling Technology	WB
Rabbit anti-p-α-syn	1:1000	23706		Cell Signaling Technology	WB, IHC
Mouse anti-Flag	1:1000	F3165		Sigma‒Aldrich	WB
Mouse anti-TH	1:1000	F-11, sc-25269		Santa Cruz Biotechnolog, Dallas, TX, USA	WB, IHC
Mouse anti-α-syn	1:1000	610787		BD Biosciences, San Jose, CA, USA	WB
Rabbit anti-microtubule-associated protein-1 LC3 II/I	1:1000	14600-1-AP		Proteintech (WuHan, China)	WB
Rabbit anti-ATP6V0A1	1:1000	NBP1-89342		Novus Biologicals (Littleton, CO, USA)	WB
HRP-conjugated anti-rabbit antibody goat anti-rabbit IgG (H+L)	1:5000	65-6120		Invitrogen	WB
HRP-conjugated anti-mouse antibody goat anti-mouse IgG (H+L)	1:5000	62-6520		Invitrogen	WB
Mouse anti-LAMP-1	1:400	ab25630	AB_470708	Abcam	ICC
Rabbit anti-LC3A/B	1:400	12741	AB_2617131	Cell Signaling Technology	ICC
Alexa Fluor 488-conjugated goat anti-rabbit IgG	1:2000	4412		Cell Signaling Technology	ICC
Alexa Fluor 555-conjugated goat anti-mouse IgG	1:2000	4409		Cell Signaling Technology	ICC
Poly-HRP Anti-Mouse/Rabbit IgG Detection System		GK600705A		Genetech, Shanghai, China	IHC

ATP6V0A1: ATPase H^+^ transporting V0 subunit A1; CTSB: cathepsin B; CTSD: cathepsin D; HRP: horseradish peroxidase; ICC: Immunocytochemistry; IHC: immunohistochemistry; LAMP-1: lysosomal-associated membrane protein 1; LAMP-1: lysosomal-associated membrane protein 1; LC3: light chain 3; mTOR: mammalian target of rapamycin; p-α-syn: phosphorylated α-synuclein; TH: tyrosine hydroxylase; WB: western blotting; α-syn: α-synuclein.

### Immunocytochemical staining

MN9D cells were seeded at a density of 30% to 40% in a confocal dish (Wuxi NEST Biotechnology Co., Ltd.) and cultured for 24 hours. Samples were washed with ice-cold PBS three times for a total of 15 minutes. They were then fixed with 4% ice-cold paraformaldehyde for 15–20 minutes, followed by three additional washes with ice-cold PBS, each lasting for 5 minutes. Subsequently, the samples were blocked with goat serum for two hours before incubating with primary antibody. The primary antibodies were incubated on cells overnight at 4°C. Subsequently, samples underwent three washes with PBS (a total of 15 minutes) and were then incubated with fluorescently labeled secondary antibodies for 1–2 hours at 37°C. Cell nuclei were stained with DAPI (F6057, Sigma–Aldrich). Images were photographed using a DM6 microscope (Leica), and the image fluorescence intensity of LC3 and lysosomal-associated membrane protein 1 (LAMP-1) was analyzed by ImageJ software (version 1.8.0). The antibodies used in immunocytochemical staining are listed in **Table 1**.

### Transmission electron microscopy

To prepare samples for transmission electron microscopy (TEM), the brains were first collected and placed in electron microscopy fixative at room temperature for 30 minutes. For tissue TEM, mice were euthanized at a dose of 60 mg/kg of 0.6% pentobarbital sodium by intraperitoneal injection (Smart), and a 2 mm × 2 mm piece of SN was rapidly excised (within 1–3 minutes). The samples were thereafter preserved with TEM fixative at 4°C for 2–4 hours. Following fixation, the specimens underwent three washes with 0.1 M phosphate buffer (PB, pH 7.4) and were subsequently post-fixed in 1% osmium tetroxide (SPI, CA, USA) with the same buffer for 2 hours at ambient temperature. The specimens were dehydrated using a sequential ethanol series (50%, 70%, 80%, 90%, 95%, and 100%), followed by 100% acetone. Next, samples were embedded in 812 resin (SPI-Pon 812 Epoxy Resin Monomer; SPI Supplies, West Chester, PA, USA) overnight at 37°C and polymerized at 65°C for 48 hours. Embedded blocks were cut into slices of 60–80 nm thickness using an ultramicrotome (UC7, Leica), and the slices stained with 2% uranyl acetate solution in saturated alcohol. Autophagosomes were examined and analyzed by TEM (Hitachi, Tokyo, Japan).

### Co-immunoprecipitation

After plating in 100-mm culture dishes, MN9D cells were separately transfected with vector and A53T-α-syn, and then cultured until around 90% confluence. Following 5-minute centrifugation at 591 × *g*, the cells were lysed using 100 μL of lysis solution containing 1% phosphatase inhibitors and 1% protease inhibitor cocktail. After incubation with lysis buffer on ice for 15 minutes, the specimens were centrifuged for 15 minutes at 4°C at 15,000 × *g*. Next, 1 μg of Protein A/G Agarose (Thermo Fisher Scientific) or 1 μg of IgG (Thermo Fisher Scientific) was added to the supernatant at a final sample volume of 500 μL. Mixes were treated with Anti-Flag (mouse, 1:1000, Cat# F3165, Sigma–Aldrich) antibody at 4°C for 14–16 hours. After reacting with Anti-Flag antibody, immunoprecipitates underwent five washes with wash buffer, after which 100 μL of elution buffer was introduced to extract protein from the magnetic beads. Subsequently, 5× loading buffer (4:1 ratio) was added to the samples without denaturation, and they were preserved at 20°C until immunoblotting. Western blot analysis was conducted according to the specified procedure.

### Real-time polymerase chain reaction

MN9D cell pellets were lysed in 1 mL TRIzol reagent (Invitrogen) at ambient temperature for 10 minutes. Then, 200 µL of RNA Extraction Auxiliary Reagent (Ecotop, GuangZhou, China) was added, followed by vortexing for 15 seconds. The mixture was subsequently centrifuged at 12,000 × *g* for 15 minutes, and the water phase layer was collected (the total volume did not exceed three-quarters). Subsequently, the samples were combined with an equivalent volume of isopropyl alcohol at –20°C overnight. The following day, the mixture was centrifuged at 800 × *g* for 5 minutes and the pellet was washed twice with 75% ethanol, dried, and then dissolved in distilled water to determine the RNA concentration.

Then, 1 μg of total RNA was converted to cDNA using a RT kit in accordance with the manufacturer’s protocol (Vazyme, Nanjing, China). In step one, a 12 µL volume containing 1 μg of RNA and 4 µL of 4 × gDNA Wiper Mix was added to tubes. After that, the mixture was reacted for 2 minutes at 42°C. Then, 4 µL of 5 × HiScript III qRT SuperMix was added to the above combination and reverse transcripted by the following conditions: 15 minutes at 37°C and 5 seconds at 85°C.

Real-time polymerase chain reaction (PCR) was performed and documented using a Bio-Rad Cx96 Detection System (Bio-Rad) to evaluate relative gene expression levels with a SYBR Green PCR kit (Vazyme). The primers used were: for mouse *ATP6V0A1*, forward 5ʹ-CGT CCC TTG GAT GCT TCT GT-3ʹ and reverse 5ʹ-TAG GCT CTT CTG CGT CCT CT-3ʹ; for mouse *SNCA*, forward 5ʹ-ACA GCA GTA GCC CAG AAG ACA G-3ʹ and reverse 5ʹ-GGC TTC AGG TTC GTA GTC TTG ATA-3ʹ; and for mouse *ACTB*, forward 5ʹ-CAT CCG TAA AGA CCT CTA TGC CAA C-3ʹ and reverse 5ʹ-ATG GAG CCA CCG ATC CAC A-3ʹ. The qPCR reaction mixture comprised 10 μL of 2× ChamQ Universal SYBR qPCR Master Mix, 0.4 μL of forward primer (10 µM), 0.4 μL of reverse primer (10 µM), and 9.2 μL of template DNA/cDNA diluted in ddH_2_O. The PCR amplification technique included an initial denaturation step at 95°C for 30 seconds, followed by 40 cycles of 95°C for 10 seconds, and 60°C for 30 seconds. Fluorescence data were corrected to ACTB expression levels, and target gene expression was determined using the 2^–∆∆Ct^ technique.

### Stereotactic injection

To determine the impact of ATP6V0A1 overexpression on α-syn-induced neurodegeneration, animals injected with AAV-ATP6V0A1 and AAV-GFP were randomly allocated into four experimental conditions: AAV-GFP + AAV-vector group, AAV-ATP6V0A1 + AAV-vector group, AAV-GFP + AAV-h-α-synA53T group, and AAV-ATP6V0A1 + AAV-h-α-synA53T group. The procedures followed were identical to those outlined earlier. In brief, AAV-vector (1.04 × 10^13^ vg/mL) and AAV-h-α-synA53T (1.26 × 10^13^ vg/mL) were co-injected with 1.5 µL of either AAV-GFP or AAV-ATP6V0A1. Behavioral assessments were conducted, and at 6 weeks post-injection, the animals were euthanized and sacrificed by decapitation.

The AAV-GFP and AAV-ATP6V0A1 vectors were constructed by ligating ATP6V0A1-encoding oligonucleotides into *NheI*/*AgeI* sites of the hSyn promoter-EGFP-MCS-SV40 PolyA (GV503) expression vector. These AAVs were produced by Shanghai Genechem Co., Ltd. For the α-syn overexpression PD model, the α-synA53T mutant was cloned into *AsisI*/*MluI* sites of the pAV-CMV-MCS-Flag vector by WZ Biosciences Inc.

The mice were placed in a stereotactic apparatus and administered an intraperitoneal dose of 60 mg/kg of 0.6% pentobarbital sodium (Smart) as an anesthetic. After making a small skin incision, 1 µL of either virus or control solution was injected into the brain parenchyma at the following coordinates (using bregma as the origin): dorsoventral, –4.7 mm; mediolateral, ±1.3 mm; and anterior–posterior, –3.0 mm. After 5 minutes, the syringe was carefully removed from the injection site. The animals’ recuperation, limb function, and feeding habits were observed after the operation.

### Behavioral tests

For all behavioral tests, the mice were transported to the experimental room 30 minutes prior to the test to allow them to acclimate to the environment. Behavioral assessments were conducted six weeks after mixed virus injection.

#### Open field test

A square arena of approximately 50 cm × 50 cm was used for the open-field test. After placing mice in the middle of this platform, their impromptu movement was observed for 15 minutes in dim illumination using a trail tracking device equipped with EthoVision XT software (Noldus Information Technology, Beijing, China). The number of entries into the core zone, total distance traveled, and movement speed were all measured and recorded. After each experiment, 70% alcohol was used to clean the box to remove any odor trails between experiments.

#### Pole-climbing test

A 50-cm long, 1 cm diameter pole covered with gauze was fastened vertically to a board. Each animal had three successful training sessions prior to the trial. After placing the mice at the top of the pole and letting them stay there for a few seconds, the experimental operator noted how long it took the mice to descend (in seconds). For further analysis, the average duration of three trials was computed for each mouse.

#### Hanging test

The mice had to grasp a horizontal wire, which was 1.5 mm in diameter, positioned 30 cm above a foam surface. The mouse’s ability to grasp the wire was then used to calculate a score based on their performance over the course of 10 seconds as follows: 2 points, the mouse grasped the wire with both forelimbs and hindlimbs; 1.5 points, the wire was held with both forelimbs and one hindlimb; 1 point, the wire was grasped with both forelimbs but no hindlimbs; and 0, fell from the wire (Chen et al., 2024b).

#### Rotarod test

The mice were trained on the rotarod (JLBehv-RRT, Shanghai, China) for 5 minutes per day at a steady speed of 10 revolutions per minute in order to acclimate them. Following three training days, the mice were put on the rotarod, which increased in speed over the course of 90 seconds, from 4 to 40 revolutions/min. The time it took for the mice to fall was noted. The test was administered three times, with a 30-minute gap between each attempt. The analysis was based on the average of the three trials.

### Immunohistochemistry

Mouse brains were fixed in 4% paraformaldehyde for 24 hours and then dried in 20% and 30% sucrose solutions. The brains were embedded in Tissue-Tek® O.C.T. Compound (SAKURA, Torrance, CA, USA) and sectioned into 15 μm thick slices using a frozen microtome (Leica). This was followed by antigen retrieval by microwaving the samples in 10 mM sodium citrate solution for 5 minutes. The tissue sections were treated with 3% hydrogen peroxide for 15 minutes to inhibit endogenous peroxidase activity. After washing three times with PBS, 1% Triton X-100 solution was used to enhance membrane permeability, facilitating improved access for antibodies to enter the cell and interact with cytoplasmic proteins. Following 1 hour blocking at room temperature with goat serum, the sections underwent incubation with primary antibodies for 14 to 16 hours at 4°C. Next, the sections were incubated for 1 hour at ambient temperature with poly-HRP Anti-Mouse/Rabbit IgG Detection System. The brain slices were then dehydrated in increasing ethanol concentrations (75%, 90%, and 100%) and treated for transparency using tissue dewaxing transparent agent. Finally, the tissue sections were treated with 3,3-diaminobenzidine (Cat# GK500710, Genetech, Shanghai, China) and imaged (Hitachi). ImageJ software was used to analyze the images. The antibodies used in immunohistochemistry are listed in **Table 1**.

### Statistical analysis

The data are presented as mean ± standard error of the mean. There were at least three biological replicates in each group. For group comparisons, the data were initially assessed to determine whether they adhered to an assumption of homogeneity of variance. One-way analysis of variance was used for three or more groups and a Tukey’s multiple comparisons test was used to compare the differences between groups. The unpaired Student’s *t*-test was used for comparisons between two groups, provided the variance was uniform. In cases where the data did not show homogeneity of variance, the Kruskal–Wallis test was used. Comparisons were considered statistically significant when the *P*-value was < 0.05. Photoshop CS6 (Adobe Systems Inc., San Jose, CA, USA), ImageJ 1.8.0, and GraphPad Prism 8.0 (version 6.07, GraphPad Software, San Diego, CA, USA) were used.

## Results

### Expression of ATPase H^+^ transporting V0 subunit A1 is downregulated in Parkinson’s disease models

**Figure 1:**
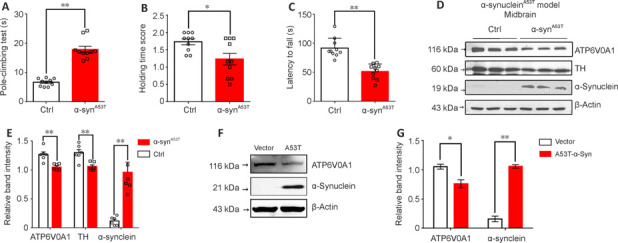
Expression of ATP6V0A1 in Parkinson’s disease models. (A) Pole-climbing time in the Ctrl and α-syn^A53T^ groups (*n* = 10). (B) Holding time scores in the grasping test for the Ctrl and α-syn^A53T^ groups (*n* = 10). (C) Latency to fall in the rotarod test for the Ctrl and α-syn^A53T^ groups (*n* = 10). (D, E) Representative western blot bands and the corresponding statistical graph of ATP6V0A1, TH, and α-syn expression in the midbrain in the α-syn^A53T^ model (*n* = 6). (F, G) Representative western blot bands and the statistical graph of ATP6V0A1 in MN9D cells transfected with α-syn virus (*n* = 3). Data are expressed as the mean ± SEM. *P < 0.05, **P < 0.01. Ctrl: Wild-type mice; α-syn^A53T^: B6. Cg-Tg (SNCA*A53T) 23Mkle/J hemizygous mice; vector: MN9D cells transduced with Ubi-MCS-3FLAG-SV40-puromycin vector; A53T: MN9D cells transduced with the A53T lentivirus. ATP6V0A1: ATPase H^+^ transporting V0 subunit A1; SNpc: Substantia nigra pars compacta; TH: tyrosine hydroxylase; α-syn: α-synuclein.

In movement tests, α-syn^A53T^ increased pole climbing time (*P* < 0.0001; **Figure 1A**), decreased holding time (*P* = 0.0153; **Figure 1B**), and reduced latency to fall (*P* < 0.0001; **Figure 1C**) compared with wild-type mice. Mice exhibited a decrease in tyrosine hydroxylase (TH) (*P* = 0.0036) and α-syn (*P* = 0.0007) expression in the midbrain of α-syn^A53T^ mice, indicating successful establishment of the PD model. ATP6V0A1 expression was also reduced in the midbrain of the α-syn^A53T^ model (*P* = 0.0017; **Figure 1D** and **E**). *In vitro* studies of the A53T mutation/α-syn overexpression (A53T-α-syn) model confirmed decreased ATP6V0A1 expression compared with the control group (*P* = 0.0208; **Figure 1F** and **G**). These findings indicate that ATP6V0A1 is linked to PD and may contribute to its pathological changes.

ATP6V0A1 knockdown and overexpressing cell models were generated using shRNA sequences and lentivirus targeting ATPase H^+^ transporting V0 subunit A1, respectively, and the knockdown or expression efficiency confirmed at the protein level (**Additional Figure 3A–E**). ATPase activity was lower in ATP6V0A1 knockdown cells compared to control cells (*P* = 0.0195; **Additional Figure 3F**).

### Transcriptomic landscape in ATPase H^+^ transporting V0 subunit A1 knockdown cells

**Figure 2:**
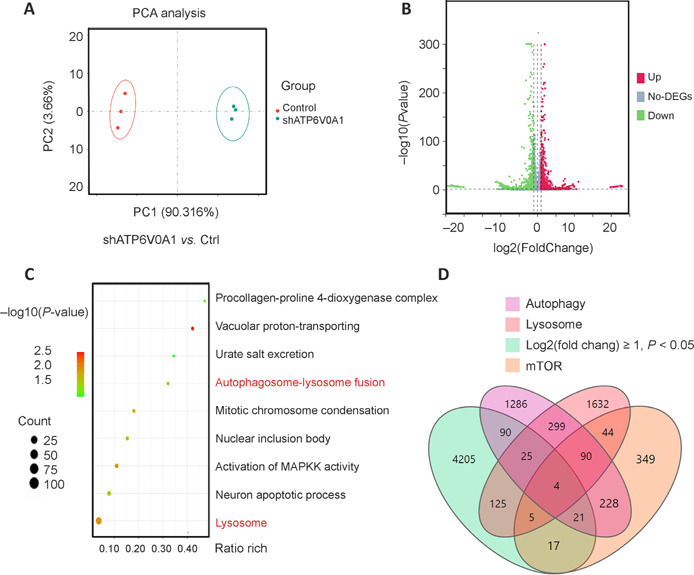
Transcriptomic profiles of knockdown cells. (A) Principal component analysis (PCA) of control and ATP6V0A1-knockdown cell lines revealed that genes were clearly separated into control and ATP6V0A1-knockdown groups. (B) DEGs are presented as a volcano plot. (C) GO enrichment bubble chart showing selected genes differentially expressed between the control and knockdown cell lines. Different genes were enriched in autophagosome‒lysosome fusion and lysosome. (D) Cross-plotting analysis of genes with *P* < 0.05 and absolute log2-fold change ≥ 1, focusing on those related to the autophagy, lysosome, and mTOR pathways in ATP6V0A1-knockdown cells. ATP6V0A1: ATPase H^+^ transporting V0 subunit A1; DEGs: differentially expressed genes; GO: Gene Ontology.

RNA sequencing was conducted on ATP6V0A1 knockdown cells to determine differences in gene expression. Control cells and ATP6V0A1 knockdown cells were clearly separated by principal component (PC) analysis, with control cells clustering on the negative side of PC1 and ATP6V0A1 knockdown cells on the positive side. This distinction accounted for 90.3% of the variation, indicating the presence of DEGs between the two groups (**Figure 2A**). A volcano plot illustrated these DEGs, highlighting genes with at least two-fold change in expression. In total, we identified 3050 DEGs, with 1333 genes upregulated and 1717 genes downregulated (**Figure 2B**). GO enrichment analysis showed that a substantial number of DEGs were enriched in lysosomal functions (**Figure 2C**). Among the 3050 DEGs identified, 140 were linked to autophagy, 159 to lysosomal processes, and 47 to the mechanistic target of rapamycin (mTOR) pathway (**Figure 2D**), underscoring a critical role of ATP6V0A1 in the autophagy–lysosomal pathway through mTOR signaling.

### ATPase H^+^ transporting V0 subunit A1 influences lysosomal function

**Figure 3:**
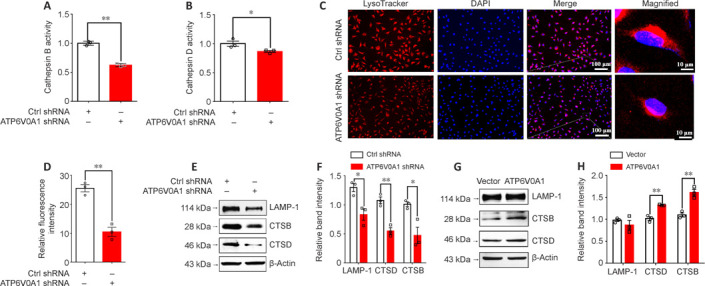
ATP6V0A1 knockdown diminished lysosome-associated functions. (A, B) CTSB and CTSD activities in Ctrl shRNA and ATP6V0A1 shRNA cells (*n* = 3). (C, D) Lysosomal acidity detected by LysoTracker probes in Ctrl shRNA and ATP6V0A1 shRNA cells, with the corresponding statistical graph of fluorescence intensity (*n* = 3). (E, F) Representative western blot bands and a statistical graph of the levels of the lysosome-related proteins LAMP-1, CTSB, and CTSD in Ctrl shRNA- and ATP6V0A1 shRNA-transfected cells (*n* = 3). (G, H) Representative western blot bands and a statistical graph of the levels of the lysosome-related proteins LAMP-1, CTSB, and CTSD in the overexpression cell lines (*n* = 3). Data are expressed as the mean ± SEM. *P < 0.05, **P < 0.01. Ctrl shRNA: MN9D cells constructed with the lentiviral vector; ATP6V0A1 shRNA: MN9D cells constructed with the knockdown sequence; Vector: MN9D cells constructed with the vector; ATP6V0A1: MN9D cells constructed with the ATP6V0A1 lentivirus. ATP6V0A1: ATPase H^+^ transporting V0 subunit A1; CTSB: cathepsin B; CTSD: cathepsin D; LAMP-1: lysosomal-associated membrane protein 1; shRNA: short hairpin RNA.

To further explore the effect of ATP6V0A1 on lysosomal function, activity of the lysosomal proteases, CTSB and CTSD, was determined using relevant activity assay kits. The activities of both enzymes were downregulated in ATP6V0A1 knockdown cells (*P* = 0.0001, **Figure 3A**; *P* = 0.0491, **Figure 3B**). Based upon the previous result showing that shATP6V0A1 decreased lysosomal acidity, we next used LysoTracker probes to qualitatively examine intracellular acidic intensity and the impact of ATP6V0A1 on pH homeostasis in MN9D cells. LysoTracker probes detected a lower number of acidic spots in ATP6V0A1 knockdown cells compared with the control group (*P* = 0.0017; **Figure 3C** and **D**). Western blot analysis further showed reduced levels of LAMP-1 (*P* = 0.0176), CTSB (*P* = 0.0213), and CTSD (*P* = 0.0028) in ATP6V0A1 knockdown cells compared to control cells (**Figure 3E** and **F**). In contrast, ATP6V0A1 overexpression increased CTSB (*P* = 0.0041) and CTSD (*P* = 0.0022) expression in MN9D cells, without causing obvious alterations in LAMP-1 expression (*P* > 0.050; **Figure 3G** and **H**). Briefly, these results confirm that ATP6V0A1 regulates physiological functions of lysosomes, including maintenance of the acidic environment and the activity and expression of lysosomal enzymes.

### ATPase H^+^ transporting V0 subunit A1 influences autophagy flux through the mechanistic target of rapamycin complex 1 signaling pathway

**Figure 4:**
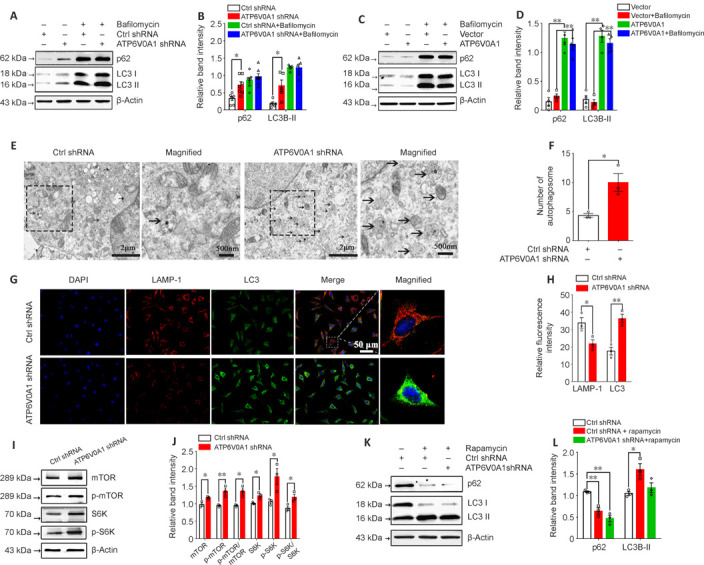
ATP6V0A1 regulates autophagy through mTORC1 signaling. (A, B) Representative western blot bands and statistical graphs showing the levels of the autophagy-related proteins LC3 and P62 in Ctrl shRNA and ATP6V0A1 shRNA cells with or without bafilomycin stimulation for 24 hours (*n* = 3). (C, D) Representative western blot bands and a statistical graph of LC3 and P62 expression in control and bafilomycin-overexpressing cells with or without bafilomycin stimulation for 24 hours (*n* = 3). (E, F) Transmission electron microscopy images of autophagosomes in MN9D cells from the control and knockdown groups (*n* = 3), with the statistical graph shown in F. The arrows show autophagosomes. (G, H) Immunocytochemical staining of LAMP-1 (red, Alexa Fluor 555) and LC3 (green, Alexa Fluor 488) upon ATP6V0A1 knockdown in MN9D cells. Magnified images of LAMP-1 and LC3 staining are shown in the right column (*n* = 3 per group). (I, J) Representative western blot bands and the statistical graph of mTORC pathway–related proteins (mTORC, p-mTORC, S6K, and p-S6K) in Ctrl shRNA and ATP6V0A1 shRNA cells. (K, L) Representative western blot bands and a statistical graph of LC3 and P62 expression in cells subjected to rapamycin treatment for 24 hours (*n* = 3). Data are expressed as the mean ± SEM. *P < 0.05, **P < 0.01. Ctrl shRNA: Lentiviral vector-transfected MN9D cells; ATP6V0A1 shRNA: lentiviral knockdown sequence-transfected MN9D cells; Vector: lentiviral vector-transfected MN9D cells; ATP6V0A1: cells constructed with ATP6V0A1 lentivirus. LAMP-1: Lysosomal-associated membrane protein 1. ATP6V0A1: ATPase H+ transporting V0 subunit A1; LAMP-1: lysosomal-associated membrane protein 1; LC3: light chain 3; mTORC1: mechanistic target of rapamycin complex 1; p-mTORC: phosphorylated mTORC; p-S6K: phosphorylated S6K; S6K: S6 kinase B1; shRNA: short hairpin RNA.

The effect of ATP6V0A1 in lysosomes suggests a critical role in autophagy. Thus, autophagic flux was investigated in ATP6V0A1 knockdown cells. ATP6V0A1 knockdown significantly increased protein levels of the polyubiquitin-binding protein, p62, and LC3II (*P* = 0.0051, *P* = 0.0146; **Figure 4A** and **B**). Conversely, no significant changes in autophagic activity were observed in overexpressing cells (**Figure 4C** and **D**). Additionally, TEM showed an increase in the amount of autophagosomes in ATP6V0A1 knockdown cells (*P* = 0.0223; **Figure 4E** and **F**). Immunofluorescence staining further corroborated these findings, demonstrating a significant decrease in LAMP-1 expression (*P* = 0.0342) and an increase in LC3 (*P* = 0.0048) in knockdown cells (**Figure 4G** and **H**). These results suggest that ATP6V0A1 may inhibit the fusion of autophagic lysosomes, thereby obstructing the autophagic process.

The mechanistic target of rapamycin complex 1 (mTORC1) is a critical protein in autophagy, and known to influence V-ATPase activity. In ATP6V0A1 knockdown cells, mTORC1 and ribosomal protein S6 kinase B1 (S6K), along with their phosphorylated forms, were activated at the basal level. Additionally, the ratio of phosphorylated (p)-mTORC1 to mTORC1 (*P* = 0.0210) and p-S6K/S6K (*P* = 0.0357) were increased in the ATP6V0A1 knockdown group, indicating activation of mTORC1-S6K signaling (**Figure 4I** and **J**). However, no significant differences were observed in p62 or LC3II expression between the two groups following stimulation with 100 nM rapamycin for 24 hours (*P* > 0.05; **Figure 4K** and **L**). Altogether, this indicates that ATP6V0A1 plays a critical role in autophagic activity through mTORC1 signaling.

### ATPase H^+^ transporting V0 subunit A1 regulates α-synuclein aggregation via the mechanistic target of rapamycin complex 1 signaling pathway

**Figure 5:**
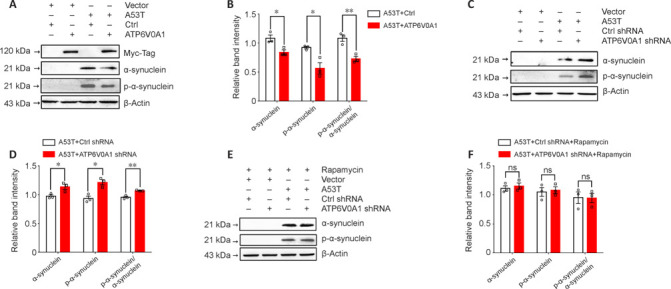
ATP6V0A1 reduces α-synuclein aggregation through autophagy and mTORC1 signaling. (A, B) Effects of ATP6V0A1 and α-synuclein lentivirus double-transfected on Myc-Tag, α-synuclein, and p-α-synuclein protein levels in MN9D cells; the statistical graph is presented on the right of panel B (*n* = 3). (C, D) Effects of double transfection with ATP6V0A1-shRNA and α-synuclein lentivirus on α-synuclein and p-α-synuclein protein levels in MN9D cells (*n* = 3). (E, F) Effects of double transfection with ATP6V0A1-shRNA and α-synuclein lentivirus on α-synuclein; p-α-synuclein protein expression in MN9D cells after rapamycin stimulation for 24 hours (*n* = 3). Data are expressed as the mean ± SEM. *P < 0.05, **P < 0.01. Vector + Ctrl: MN9D cells were transfected with a lentiviral vector and Ctrl vector; Vector + ATP6V0A1: MN9D cells were transfected with a lentiviral vector and ATP6V0A1 lentivirus; A53T + Ctrl: MN9D cells were transfected with A53T lentivirus and Ctrl vector; A53T + ATP6V0A1: MN9D cells constructed with A53T lentivirus and ATP6V0A1 lentivirus; Vector + Ctrl shRNA: lentiviral vector and ATP6V0A1 knockdown vector-transfected MN9D cells; Vector + ATP6V0A1 shRNA: lentiviral vector and lentivirus-mediated knockdown sequence-transfected MN9D cells; A53T + Ctrl shRNA: A53T-overexpressing lentivirus and lentiviral vector-transfected MN9D cells; A53T + ATP6V0A1 shRNA: A53T-overexpressing lentivirus and ATP6V0A1-knockdown lentivirus-transfected MN9D cells. ATP6V0A1: ATPase H+ transporting V0 subunit A1; ns: not significant; mTORC1: mechanistic target of rapamycin complex 1; p-: phosphorylated; shRNA: short hairpin RNA.

The autophagy–lysosomal pathway is involved in long-lived protein degradation. In the A53T-α-syn induced PD model, ATP6V0A1 overexpression ameliorated the levels of α-syn (*P* = 0.0264) and p-α-syn proteins (*P* = 0.0228). Moreover, the ratio of p-α-syn/α-syn decreased in ATP6V0A1 overexpressing cells (*P* = 0.0074, **Figure 5A** and **B**). In contrast, α-syn (*P* = 0.0166) and p-α-syn (*P* = 0.0101) protein levels increased in ATP6V0A1 knockdown cells compared to control cells; there was also an increased ratio of p-α-syn/α-syn (*P* = 0.0033; **Figure 5C** and **D**). However, α-syn did not directly bind to ATP6V0A1 (**Additional Figure 4A**), and neither ATP6V0A1 knockdown nor overexpression affected α-syn mRNA levels (*P* > 0.05; **Additional Figure 4B** and **C**). Given that ATP6V0A1 knockdown reduces autophagic activity through mTORC1 signaling, we further investigated whether α-syn levels were influenced by mTORC1 signaling. No apparent changes in p-α-syn and α-syn expression or p-α-syn/α-syn ratio were detected in double transfected ATP6V0A1 knockdown cells after rapamycin stimulation, suggesting that ATP6V0A1 regulates α-syn levels through its effects on mTORC1 (*P* > 0.05; **Figure 5E** and **F**).

### ATPase H^+^ transporting V0 subunit A1 alleviates AAV-h-α-syn^A53T^-induced Parkinson’s disease-like symptoms and pathology

**Figure 6:**
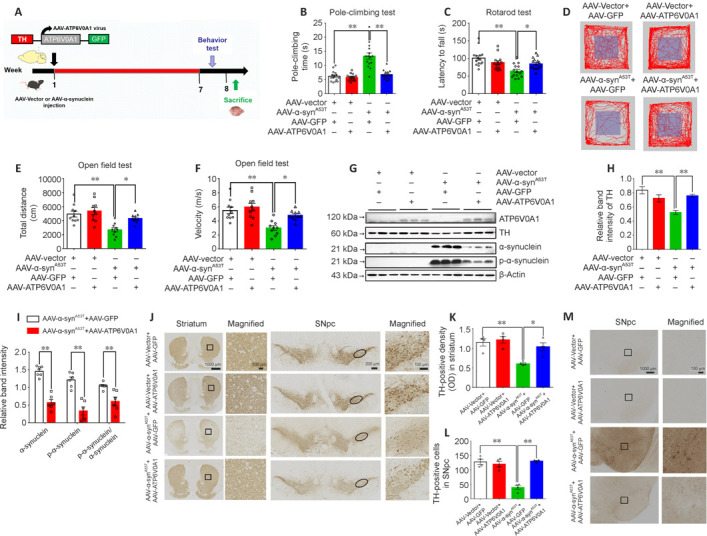
ATP6V0A1 overexpression in the SNpc alleviates motor dysfunction and dopaminergic neuron degeneration in the α-synuclein model. (A) Flowchart of the experimental procedure for the α-synuclein model. (B) Pole-climbing time in the following groups: AAV-GFP + AAV-vector, AAV-ATP6V0A1 + AAV-vector, AAV-GFP + AAV-h-α-syn^A53T^, and AAV-ATP6V0A1 + AAV-h-α-syn^A53T^ (*n* = 14–16). (C) Latency to fall in the rotarod test for the groups mentioned above (*n* = 14–16). (D) Representative path tracings in the open-field test (*n* = 10). (E, F) Total distance traveled and velocity of the open-field test in the following groups: AAV-GFP + AAV-vector, AAV-ATP6V0A1 + AAV-vector, AAV-GFP + AAV-h-α-syn, and AAV-ATP6V0A1+AAV-h-α-syn^A53T^ (*n* = 10). (G–I) Representative western blot bands and statistical graphs of ATP6V0A1, TH, α-synuclein, and p-α-synuclein in midbrain tissue from each group (*n* = 6). (J–L) Representative images and statistical graphs of immunohistochemical staining for TH in the striatum and SNpc in each group (*n* = 4). (M) Representative images of immunohistochemical staining for p-α-synuclein in the SNpc of each group (*n* = 3). Data are presented as the mean ± SEM. *P < 0.05, **P < 0.01. AAV-GFP + AAV-vector: Mice were injected with AAV-GFP or AAV-vector. AAV-ATP6V0A1 + AAV-vector: Mice were stereotactically injected with AAV-ATP6V0A1 or the vector. AAV-GFP + AAV-h-α-synA53T: Mice were injected with AAV-GFP and AAV-h-α-synA53T; AAV-ATP6V0A1 + AAV-h-α-syn^A53T^: Mice were injected with AAV-ATP6V0A1 and AAV-h-α-syn^A53T^. AAV: Adeno-associated virus; ATP6V0A1: ATPase H+ transporting V0 subunit A1; GFP: green fluorescent protein; h-α-syn: human α-synuclein; SNpc: substantia nigra pars compacta; TH: tyrosine hydroxylase; α-syn: α-synuclein.

To further examine the influence of ATP6V0A1 on α-syn expression, we generated a mouse model by targeting the bilateral substantia nigra pars compacta (SNpc) with AAV-h-α-syn^A53T^ and AAV-ATP6V0A1. The mice were raised for 6 weeks before testing (**Figure 6A**). In movement tests, mice receiving AAV-h-α-syn^A53T^ combined with AAV-GFP exhibited increased pole climbing time (*P* < 0.0001; **Figure 6B**) and reduced latency to fall (*P* < 0.0001; **Figure 6C**), with the control group used as a reference. Conversely, the combination of AAV-h-α-syn^A53T^ and AAV-ATP6V0A1 reversed these effects, restoring pole climbing time (*P* = 0.0022; **Figure 6B**) and improving performance on the rotarod test (*P* = 0.0257; **Figure 6C**). Additionally, the grip strength test detected reduced limb strength in mice treated with AAV-h-α-syn^A53T^ combined with AAV-GFP (*P* = 0.0229; **Additional Figure 5A**), yet ATP6V0A1 overexpression did not rescue this deficit (*P* > 0.05; **Additional Figure 5A**). No significant differences were found in the grasping test among the four groups (*P* > 0.05; **Additional Figure 5B**). Representative path tracings of the open-field test are shown in **Figure 6D**. Mice treated with AAV-h-α-syn^A53T^ demonstrated decreased total distance traveled (*P* = 0.0006; **Figure 6E**) and reduced movement velocity (*P* < 0.001; **Figure 6F**). Treatment with AAV-ATP6V0A1 improved both traveled distance (*P* = 0.0171; **Figure 6E**) and velocity (*P* = 0.0130; **Figure 6F**). Regarding the time mice spent in the center zone, the AAV-ATP6V0A1 group spent less time in this zone (*P* = 0.0079; **Additional Figure 5C**). Both AAV-h-α-syn^A53T^+AAV-GFP and AAV-h-α-syn^A53T^+AAV-ATP6V0A1 significantly decreased the number of mouse entries to the center zone in comparison with the reference group (*P* = 0.0007; **Additional Figure 5D**). However, no differences were noted in terms of residence time in the middle area or the number of entries to the center zone between the AAV-h-α-syn^A53T^+AAV-GFP and AAV-h-α-syn^A53T^+AAV-ATP6V0A1 groups (*P* > 0.05; **Additional Figure 5C** and **D**). In general, ATP6V0A1 appears to mitigate the movement disorders observed in α-syn^A53T^ mice.

Furthermore, compared to the AAV-h-α-syn^A53T^ group, AAV-h-α-syn^A53T^ in conjunction with AAV-ATP6V0A1 significantly reduced α-syn (*P* < 0.0001) and p-α-syn (*P* = 0.0001) protein levels, the ratio of p-α-syn/α-syn (*P* = 0.0066), and increased TH protein levels (*P* = 0.0025) in the midbrain (**Figure 6G–I** and **M**). Moreover, AAV-h-α-syn^A53T^ combined with AAV-ATP6V0A1 rescued AAV-h-α-syn^A53T^-induced TH loss in both the striatum (*P* = 0.0119) and SNpc (*P* < 0.0001; **Figure 6J–L**). These results suggest that overexpression of ATP6V0A1 in SNpc DA neurons mitigates the PD phenotypes induced by α-syn^A53T^.

## Discussion

Our research has identified ATP6V0A1 as a crucial factor in PD development. A significant decrease in ATP6V0A1 expression was observed both *in vitro* and *in vivo*. Furthermore, ATP6V0A1-shRNA increased pathological α-syn levels by impairing lysosomal function and inhibiting autophagy through the mTORC pathway. In contrast, ATP6V0A1 overexpression alleviated levels of α-syn and p-α-syn proteins, mitigated the degeneration of DA neurons, and improved motor dysfunction, indicating a protective role in PD.

V-ATPase is a proton-pumping enzyme that transports H^+^ into intracellular compartments to facilitate ATP hydrolysis (Futai et al., 2019). V-ATPase influences lysosome function by regulating lysosomal pH (4.5–5.0) and maintaining hydrolase activity (Maxson and Grinstein, 2014). ATP6V0A1 is the V0 domain subunit of V-ATPase, and is highly expressed in the brain. Missense variants of *ATP6V0A1*, specifically R741Q, A512P, and N534D, are associated with lysosomal acidification disorders, which manifest in affected individuals as intellectual disability, developmental delay, and epilepsy, indicating that ATP6V0A1 may play a significant role in the brain by regulating lysosomal function (Aoto et al., 2021). In *Drosophila*, neurons with V-ATPase V0a1 loss or mutation exhibit increased susceptibility to amyloid beta and tau neurotoxicity (Williamson and Hiesinger, 2010). In presenilin 1 knockout cells, N-glycosylation of the V0a1 subunit was disrupted, resulting in further deposition of pathogenic proteins and neuronal destruction in AD patients (Lee et al., 2010). Considering the similar pathological mechanisms underlying PD and AD, ATP6V0A1 may play a significant role in PD development. Wallings et al. (2019) found that both hWT-LRRK2 and LRRK2-G2019S can bind to ATP6V0A1, leading to decreased expression and mislocalization of V0a1. However, the precise mechanism between PD and *ATP6V0A1* remains obscure (Colacurcio and Nixon, 2016). Our previous research involving 649 PD patients and 533 healthy controls found that the *ATP6V0A1* rs601999 CC genotype was associated with PD (Chen et al., 2019). However, the rs601999 CC variant is located in an intron of chromosome 17 and may not significantly influence ATP6V0A1 protein levels. ATP6V0A1 R741Q, A512P, and N534D are reportedly significant for brain development and epilepsy. Along with high expression levels of ATP6V0A1 in the brain, this suggests that ATP6V0A1 plays a crucial role in regulating brain physiological functions. In our study, ATP6V0A1 expression was decreased in the α-syn overexpression model. While no studies have directly investigated the impact of α-syn on ATP6V0A1, the observed reduction in ATP6V0A1 levels in these PD models may be attributed to lysosomal membrane impairment and deacidification caused by α-syn (Bourdenx et al., 2016; Dong et al., 2019; Bellomo et al., 2020; Ge et al., 2021). Given that V-ATPase is abundantly expressed in lysosomes and other organelles, our findings underscore its potential role in PD pathology, particularly with respect to lysosome function (Hernandez et al., 2010). We observed that ATP6V0A1 knockdown cells exhibited decreased activity and expression of the lysosomal enzymes, CTSB and CTSD, while overexpression of ATP6V0A1 led to an increase in their expression. Consistently, lysosomal acidification was disrupted and the lysosomal marker LAMP-1 was decreased, although no significant differences were detected in ATP6V0A1 overexpressing cells. Our results demonstrate that ATP6V0A1 plays a crucial role in lysosomal function, with its deficiency leading to lysosomal dysfunction. Our findings are consistent with those of Hinton et al. (2009), who observed increased pH of endosomes/lysosomes following the knockdown of a1, a2, and a3 in human breast cancer cells. Furthermore, it has been demonstrated that ATP6V0A1 promotes the maturation of lysosomal endosomes via the Wnt pathway. Nevertheless, in neural crest cells, ATP6V0A1 deficiency impaired endosome maturation without affecting acidification, indicating that ATP6V0A1 plays distinct roles in various aspects of lysosomal function through different pathways (Tuttle et al., 2014).

Autophagy is essential for maintaining intracellular homeostasis, facilitating autophagosome formation, the engulfment of long-lived proteins or organelles, and the fusion of autophagosomes with lysosomes (Corti et al., 2020; Lizama and Chu, 2021; Zhao et al., 2025). Autophagy is particularly significant in neurodegenerative diseases and accounts for pathological protein degradation (Martini-Stoica et al., 2016). The gene encoding α-syn (*SNCA*) is recognized as a virulence factor in PD, with the aberrant deposition of α-syn implicated in the continuous degeneration of DA neurons in the SN (Zhang et al., 2022). The clearance of α-syn is heavily reliant on both the ubiquitin–proteasome system and the autophagy–lysosomal pathway (Xilouri et al., 2013). Inhibition of the autophagy–lysosomal pathway triggers the release of toxic α-syn through extracellular vesicles, which can potentially effect the formation of α-syn aggregation (Sahoo et al., 2022). Notably, in a rotenone-induced model, knockdown of ATP6V0A1 suppressed rifampicin-mediated autophagosome–lysosome fusion (Liang et al., 2020). It has been shown that ATP6V0A1 knockdown causes the accumulation of autophagosomes and impaired autophagosome–lysosome fusion in lung cancer cells (Hsin et al., 2012). In retinal pigment epithelium cells, ATP6V0A1 interacts with CRYBA1 to regulate endolysosomal acidification, thereby controlling phagocytosis and autophagy (Valapala et al., 2014). Our findings indicate that in ATP6V0A1 knockdown cells, autophagic flux is inhibited, as shown by increased levels of LC3II and p62 in comparison with the control group. However, the two groups exhibited no significant differences following bafilomycin stimulation, likely due to the action of bafilomycin as a V-ATPase blocker and autophagy inhibitor. Our results are consistent with previous research, suggesting that ATP6V0A1 plays a significant role in autophagy.

The mTORC1 signaling pathway is a critical regulator of autophagy and regulates various stages of autophagy (Noda, 2017). Our results indicate that ATP6V0A1 plays a significant role in autophagy; however, it is uncertain whether this effect is mediated by the mTORC1 signaling pathway. Thus, we examined mTORC1 signaling pathway activity, finding that it was activated in ATP6V0A1 knockdown cells, as shown by increased expression levels of mTORC1, p-mTORC1, S6K, and p-S6K. Importantly, autophagy inhibition was reversed by rapamycin, an autophagy agonist, thereby confirming that ATP6V0A1 mediates autophagy through the mTORC1 pathway. However, Mo et al., (2025) found that ATP6V1B1 regulated activation of the mTOR/autophagy pathway and that ATP6V1B1 knockdown decreased p-mTOR protein levels, contradicting our findings (Mo et al., 2024). Currently, there are no relevant reports regarding regulation of the mTORC pathway by ATP6V0A1. The discrepancy between our results and those of Mo et al., may be attributed to the fact that ATP6V1B1 is primarily located in the V-ATPase V1 region and is involved in ATP hydrolysis, whereas ATP6V0A1 is situated in the V0 region, which is primarily responsible for H^+^ transport. Considering the critical role of autophagy in the degradation of α-syn, we subsequently examined α-syn expression and found that ATP6V0A1 knockdown caused increased levels of pathological α-syn. Conversely, ATP6V0A1 overexpression ameliorated pathological α-syn levels in an A53T-α-syn-induced model. The results obtained from our double transfection model are consistent with those reported by Chen et al. (2020), who transfected SHSY5Y cells with recombinant human anti-SNCA antibody (NAC32) and α-syn plasmid. Intriguingly, rapamycin reversed the abnormal pathological levels of α-syn, indicating that ATP6V0A1 knockdown can lead to autophagy dysfunction, which in turn results in increased α-syn protein levels.

To further examine the role of ATP6V0A1 *in vivo*, we stereotactically injected AAV-ATP6V0A1 and AAV-h-α-syn^A53T^ simultaneously into 8-week-old mice. Our results showed that ATP6V0A1 overexpression ameliorated behavioral impairments in α-syn-induced mice, decreased p-α-syn levels, and alleviated DA neuron loss. Conversely, ATP6V0A1 knockdown impaired lysosomal function, disrupted lysosome-related autophagy, and increased pathological α-syn protein levels. This increase in α-syn consequently exacerbated lysosomal damage and aggravated autophagy dysfunction, hindering α-syn clearance and compromising lysosomal integrity. Nonetheless, ATP6V0A1 overexpression effectively enhanced lysosomal enzyme activity and improved motor performance, highlighting its potential to mitigate α-syn-induced damage and protect DA neurons *in vivo*. In addition to its effects on lysosomal function, the overexpression of ATP6V0A1 also mitigated PD-like phenotypes, evidenced by increased TH expression, reduced pathological α-syn levels, and improved behavioral outcomes in α-syn-induced mouse models. Although the role of ATP6V0A1 in PD has not been reported, ATP6V0A1 was previously investigated to determine how to reduce levels of the pathological protein amyloid beta protein precursor in AD, which was consistent with our observation that ATP6V0A1 can decrease α-syn protein levels. This suggests that ATP6V0A1 has a specific protective effect against neurodegenerative diseases. Notably, no significant differences in TH expression were observed *in vitro*, suggesting that ATP6V0A1 may exert an indirect influence on TH expression. While MN9D cells (which express TH) have been extensively used as *in vitro* models for studying DA neurons, replicating DA release and transport *in vitro* remains challenging (Zhang et al., 2020).

Nevertheless, our study has several limitations that should be noted. This includes our inability to further investigate other ATP6V0A1 variants in clinical settings, such as R741Q or A512P, which have been linked to brain development and the risk of PD. In our basic research, we used A53T adenovirus and ATP6V0A1 adenovirus overexpression, but were unable to confirm the impact of ATP6V0A1 on α-syn in two-gene hybrid mice. Mechanistically, we focused on lysosomal autophagy, but could not validate this in animal models. These limitations mean that we could not fully explore the role of ATP6V0A1 in PD. Therefore, future studies will involve whole genome sequencing of ATP6V0A1 using the original clinical database to further explore the relationship between ATP6V0A1 and PD risk. Additionally, we need further research on lysosomal function, lysosomal autophagy, and α-syn degradation in two-gene hybrid mice. We anticipate that ATP6V0A1 may be a potential biomarker for PD.

In conclusion, our data suggests that ATP6V0A1 protects against PD-like pathophysiology through autophagy, thereby reducing pathological α-syn protein levels, alleviating DA neuronal degeneration, and improving behavior performance. These findings highlight the neuroprotective role of ATP6V0A1 and its possible function as a novel target for PD treatment.

## Additional files:

***Additional Figure 1:*** *Flowchart of the experiments.*

Additional Figure 1 Flowchart of the experiments.
ATP6V0A1: ATPase H+ transporting V0 subunit A1; PD: Parkinson’s disease; TH: tyrosine hydroxylase; α-syn: α-synuclein.

***Additional Figure 2:*** *Experimental timeline.*

Additional Figure 2 Experimental timeline.
AAV: Adeno-associated virus; ATP6V0A1: ATPase H+ transporting V0 subunit A1; GFP: green fluorescent protein; PD: Parkinson’s disease; TH: tyrosine hydroxylase.

***Additional Figure 3:*** *ATP6V0A1-knockdown and ATP6V0A1-overexpressing cell model construction and ATPase activity in ATP6V0A1-knockdown cells.*

Additional Figure 3 ATP6V0A1-knockdown and ATP6V0A1-overexpressing cell model construction and ATPase activity in ATP6V0A1-knockdown cells.
(A) Relative mRNA expression of *ATP6V0A1* after transfection with three knockdown sequences (*n* = 3). (B, C) Representative western blot bands and statistical graph of ATP6V0A1 and TH expression in ATP6A1-knockdown cell lines (*n* = 3). ATP6V0A1 expression was decreased in the ATP6V0A1 shRNA group. (D, E) Representative western blot bands and a statistical graph of ATP6V0A1 and TH expression in ATP6V0A1-overexpressing cell lines (*n* = 3). ATP6V0A1 expression was increased in the ATP6V0A1 group. (F) ATPase activity in control and ATP6V0A1-knockdown cells. ATPase activity was decreased in the ATP6V0A1 shRNA group. All the data are expressed as the mean ± SEM. *P < 0.05, **P < 0.01. Ctrl shRNA: MN9D cells constructed with the vector; ATP6V0A1 shRNA: MN9D cells constructed with the knockdown sequence; Vector: MN9D cells constructed with the vector; ATP6V0A1: MN9D cells constructed with the ATP6V0A1 lentivirus. ATP6V0A1: ATPase H+ transporting V0 subunit A1; shRNA: short hairpin RNA; TH: Tyrosine hydroxylase.

***Additional Figure 4:*** *Effect of ATP6V0A1 knockdown on α-synuclein.*

Additional Figure 4 Effect of ATP6V0A1 knockdown on α-synuclein.
(A) Co-immunoprecipitation showed that ATP6V0A1 could not bind directly to α-synuclein. (B, C) Relative mRNA expression of α-synuclein in ATP6V0A1-knockdown cells or ATP6V0A1-overexpressing cells (*n* = 3); statistical significance was determined by Student's *t*-tests. The data are expressed as the mean ± SEM. ATP6V0A1: ATPase H+ transporting V0 subunit A1.

***Additional Figure 5:*** *Behavioral tests of ATP6V0A1 overexpression in the h-α-syn*^*A53T*^ *model.*

Additional Figure 5 Behavioral tests of ATP6V0A1 overexpression in the *h*-α-synA53T model.
(A) Grip strength in the AAV-GFP + AAV-vector, AAV-ATP6V0A1 + AAV-vector, AAV-GFP+AAV-*h*-α-synA53T, and AAV-ATP6V0A1+AAV-*h*-α-synA53T groups (*n* = 14–16). (B) Holding time scores in the grasping test for the AAV-GFP+AAV-vector, AAV-ATP6V0A1+AAV-vector, AAV-GFP+AAV-*h*-α-synA53T, and AAV-ATP6V0A1+AAV-*h*-α-synA53T groups (*n* = 14–16). (C) Duration in the center zone of the open-field test for the AAV-GFP+AAV-vector, AAV-ATP6V0A1+AAV-vector, AAV-GFP+AAV-*h*-α-synA53T, and AAV-ATP6V0A1+AAV-*h*-α-synA53T groups (*n* = 10). (D) Number of entries in the center zone of the open-field test for the AAV-GFP+AAV-vector, AAV-ATP6V0A1+AAV-vector, AAV-GFP+AAV-*h*-α-synA53T, and AAV-ATP6V0A1+AAV-*h*-α-synA53T groups (*n* = 10). The data are expressed as the mean ± SEM. *P < 0.05, **P < 0.01. AAV-GFP+AAV-vector: Mice were injected with AAV-GFP or AAV-vector. AAV-ATP6V0A1+AAV-vector: Mice were stereotactically injected with AAV-ATP6V0A1 or the vector. AAV-GFP+AAV-*h*-α-synA53T: Mice were injected with AAV-GFP and AAV-*h*-α-synA53T; AAV-ATP6V0A1+AAV-*h*-α-synA53T: Mice were injected with AAV-ATP6V0A1 and AAV-*h*-α-synA53T. AAV: Adeno-associated virus; ATP6V0A1: ATPase H+ transporting V0 subunit A1; GFP: green fluorescent protein; *h*-α-syn: human α-synuclein; α-syn: α-synuclein.

## Data Availability

*All relevant data are within the paper and its Additional files.*
